# Toxic Metal Concentrations in Cigarettes Obtained from U.S. Smokers in 2009: Results from the International Tobacco Control (ITC) United States Survey Cohort

**DOI:** 10.3390/ijerph110100202

**Published:** 2013-12-20

**Authors:** Rosalie V. Caruso, Richard J. O’Connor, W. Edryd Stephens, K. Michael Cummings, Geoffrey T. Fong

**Affiliations:** 1 Department of Health Behavior, Roswell Park Cancer Institute, Elm and Carlton Streets, Buffalo, NY 14263, USA; E-Mail: Rosalie.Caruso@RoswellPark.org; 2 Department of Earth & Environmental Sciences, University of St Andrews, Irvine Building, North Street, St Andrews, Fife, KY16 9AL, UK; E-Mail: wes@st-andrews.ac.uk; 3 Department of Psychiatry and Behavioral Sciences, Medical University of South Carolina, Charleston, SC 29425, USA; E-Mail: cummingk@musc.edu; 4 Department of Psychology, University of Waterloo, Waterloo, ON, N2L 3G1, Canada; E-Mail: geoffrey.fong@uwaterloo.ca; 5 Ontario Institute for Cancer Research, Toronto, ON, M5G 0A3, Canada

**Keywords:** metals, toxicity, tobacco, smoking

## Abstract

Smoking-related diseases can be attributed to the inhalation of many different toxins, including heavy metals, which have a host of detrimental health effects. The current study reports the levels of arsenic (As), cadmium (Cd), chromium (Cr), nickel (Ni), and lead (Pb) in cigarettes obtained from adult smokers participating in the 2009 wave of the ITC United States Survey (N = 320). The mean As, Cd, Cr, Ni, and Pb levels were 0.17, 0.86, 2.35, 2.21, and 0.44 µg/g, respectively. There were some differences in metal concentrations of cigarette brands produced by different manufacturers, suggesting differences in the source of tobaccos used by different companies. For Ni, there were significant pairwise differences between Philip Morris U.S. (PMUSA) and R.J. Reynolds (RJR) brands (PMUSA higher; p < 0.001), PMUSA and other manufacturer (OM) brands (PMUSA higher; p < 0.001), and RJR and OM brands (RJR higher; p = 0.006). For Cr, RJR brands had higher levels than did OM brands (p = 0.02). Levels of As, Cd, and Pb did not differ significantly across manufacturer groups (p > 0.10). Because of the variety of toxic heavy metals in cigarette tobacco, and their numerous negative health effects, metal content in cigarette tobacco should be reduced.

## 1. Introduction

Cigarette smoking leads to an estimated 443,000 deaths per year in the United States [1]. Smoking-related diseases are ultimately the result of nicotine addiction [2], which leads to the repeated inhalation of a variety of toxicants in cigarette smoke, including nitrosamines, polycyclic aromatic hydrocarbons, volatile organic compounds, and several toxic heavy metals [3]. Among these toxicants, there have been comparatively fewer studies conducted on the role of heavy metals as causes of smoking-related diseases and there is a need for basic studies on the levels of heavy metals in cigarettes and other tobacco products.

The presence of trace amounts of metals in tobacco smoke has been known for some time [4]. Those most commonly associated with health effects include arsenic (As), cadmium (Cd), chromium (Cr), nickel (Ni), and lead (Pb). As, Cd, and Cr (VI), as well as Ni compounds, are all designated as carcinogenic to humans by the International Agency for Research on Cancer [5]. As and Cd also have established non-cancer toxicities, including the cardiovascular and renal systems [6]. Pb is a Class 2B carcinogen, and toxic to humans, in particular affecting the nervous system and (in youth) neurodevelopment [6,7]. Fresquez *et al.* [8] have recently reported reference values for major U.S. cigarette brands, with As ranging from 0.22–0.36 μg/g, Cd from 1.0–1.7 μg/g, Cr from 1.4–3.2 μg/g, Ni from 2.1–3.9 μg/g, and Pb from 0.6–1.2 μg/g dry tobacco. Some components transfer better into smoke than others, ranging from near 1% (As) to as high as 22% (Cd) [4,9,10,11], although it should be noted that because there is currently no accepted standard for the measurement of transfer rates, their usefulness for understanding differences across metals is lowered. 

The level of exposure to metals in the smoke drawn from a single cigarette is small and likely not acutely toxic, but the accumulation of metals in the body over months, years, and decades of exposure is, depending on clearance rates, a health concern [6,12]. Several heavy metals found in tobacco smoke, such as Cd, Cr, Pb, and Ni, accumulate in tissues and fluids after smoking [13,14,15,16]. This is a particular issue for cadmium (Cd) and lead (Pb), which have long (10–12 year) half-lives in the human body. Cigarette smoking is a major exposure route for Cd (and to a lesser extent Pb) in the general population [15,16]. 

Biomonitoring studies show that smokers have substantially higher Cd and Pb levels [15,16,17], and bioaccumulation of metals has also been demonstrated in those chronically exposed to tobacco smoke pollution (also known as second-hand smoke) [15,16,18]. Because the use of arsenic-containing pesticides has declined, smoking no longer appears to represent a major exposure pathway for As [19]. Human toxicity of As is made complex by its multiple valence states and this has not yet been satisfactorily characterized in tobacco smoke, although it is known that As exists in mainstream smoke dominantly in its more toxic inorganic forms [20], making it a noteworthy element to examine. 

Viana and colleagues [21] noted substantial cross-manufacturer variability in levels in Brazilian cigarettes, particularly for Philip Morris-produced brands, suggesting sourcing of tobacco may play a role. Metals enter the tobacco plant during growing dominantly by absorption from soil and from the application of fertilizers, with uptake rate influenced by soil pH [4,12]. Cigarettes made from tobacco grown in China have been found to have elevated levels of lead and cadmium compared to cigarettes made from tobacco grown elsewhere, likely the result of soil contamination due to environmental pollution [22,23,24]. Other potential sources of metals in finished cigarettes include pesticides and additives such as flavorants or humectants applied to products during the manufacturing process [25]. 

The U.S. Food and Drug Administration (FDA), now charged with regulation of tobacco products sold in the United States, has raised concerns about metals found in tobacco products [26,27]. In March 2012, the FDA issued draft guidance on reporting harmful and potentially harmful constituents (HPHC) in tobacco products and listed several metals (including As, Cd, Cr, Ni, and Pb) as compounds for reporting [28]. Understanding the profile of heavy metals in the current U.S. market is complicated by the fact that much of the tobacco used in cigarettes sold in the U.S. comes from overseas markets [29]. The current study reports on the levels of As, Cd, Cr, Ni, and Pb in cigarettes obtained from a subset of participants in the 2009 wave of the International Tobacco Control (ITC) United States Survey, which includes a national sample of U.S. adult cigarette smokers [30]. 

## 2. Experimental Section

The cigarette packs analyzed in this study came from wave 7 of the International Tobacco Control (ITC) United States Survey, a longitudinal survey that has been conducted among a nationally representative cohort of U.S. adult (≥18 years) smokers approximately annually since 2002. As part of the wave 7 survey, smokers were asked to supply a fresh pack of their cigarettes for analysis. For a description of the ITC survey and pack collection method, see Fong *et al.* [31], Thompson *et al.* [32], Fix *et al.* [30], and O’Connor *et al.* [33].

In this study, 320 cohort participants returned an unopened pack of their usual brand of cigarettes to the ITC research team at Roswell Park Cancer Institute, where they were catalogued, placed in −20 °C storage until testing, and conditioned to 22 °C and 60% relative humidity prior to testing for physical characteristics. Cigarette design testing procedures followed previously published methods [22,34,35,36]. Per-cigarette tobacco weight and moisture content were determined as the average of five sticks using a halogen moisture analyzer (HR-83, Mettler-Toledo, Columbus, OH, USA). Ten sticks were then chosen at random from each pack, placed in polypropylene zip-top bags with code numbers, and sent to the University of St. Andrews, Scotland for the metals analysis. 

To quantify the metal concentrations, the tobacco was removed from the cigarettes and dried for 48 hours before pulverising to powder in a Rocklabs benchtop mill using a tungsten carbide pot. Pellets were pressed from the powder at 20 tons pressure. Polarized energy dispersive x-ray fluorescence (XRF) was used to measure the concentrations of 25 elements (Mg, Al, Si, P, Cl, S, K, Ca, Ti, Cr, Mn, Fe, Ni, Cu, Zn, As, Br, Rb, Sr, Zr, Nb, Cd, Sn, Ba, Pb) in these pellets using an established method [37]. Analysis was carried out using a Panalytical Epsilon 5 XRF with Gd X-ray tube. Two sigma (2σ) errors for the elements of interest in this paper are 0.54 μg·g^−1^ for Cr, 0.48 µg·g^−1^ for Ni, 0.14 µg·g^−1^ for As, 0.30 µg·g^−1^ for Cd and 0.28 µg·g^−1^ for Pb. Limits of detection for these metals are 0.31, 0.14, 0.15, 0.24 and 0.19 respectively (all in µg·g^−1^). Analysis for these metals and metalloids was completed on 315 cigarette packs. 

Data analysis was completed using SPSS Version 21.0 (IBM, Armonk, NY, USA). Concentrations are reported per microgram of tobacco (dry weight) for the five metals of focus in this study (As, Cd, Cr, Ni, Pb). Metal concentration averages were reported across all brands, and separately by manufacturer (Philip Morris USA, RJ Reynolds, Other Manufacturers). Analysis by brand characteristics (menthol, light/mild descriptor, tobacco weight, moisture) used a GEE approach to account for multiple instances of the same brand style (using UPC as a ‘subject ID’). Generalized linear model analyses looked for patterns in toxic metal concentrations by participant age, gender, income level, and education level, stratified by product manufacturer. Bonferroni *post-hoc* tests were used to conduct paired comparisons with multi-level factors as appropriate. 

## 3. Results and Discussion

**Figure 1:**
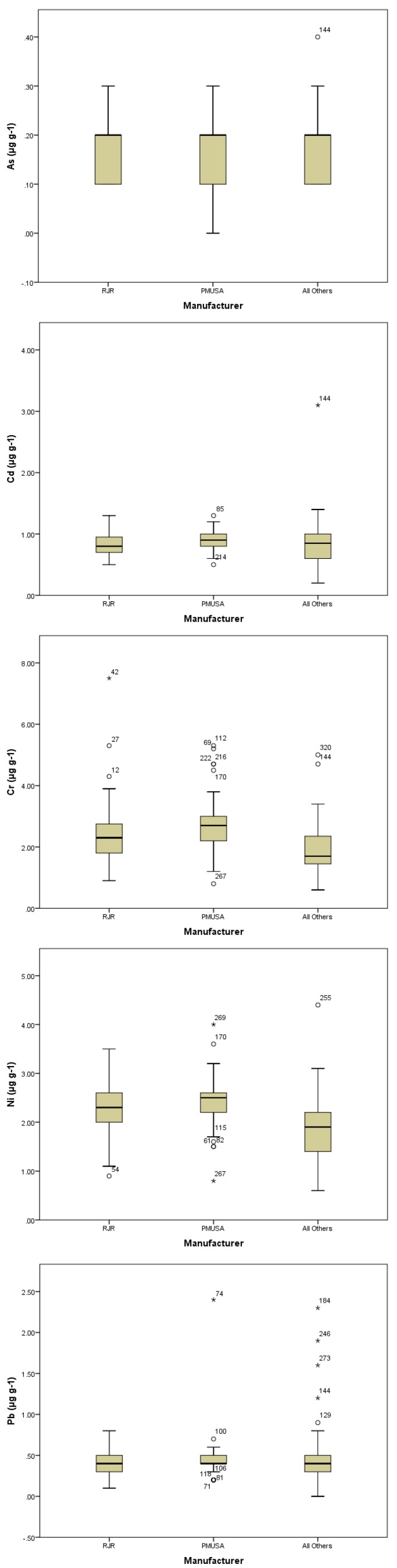
Boxplots of metal concentrations by manufacturer.

On average, cigarettes contained 0.62 g tobacco (dry weight; SD 0.08, range 0.42–1.01 g) and had a moisture level of 15.0% (SD 1.1, range 11.8–18.2 g). Table 1 presents the mean, standard deviation, and range of each metal across the sample in micrograms per gram tobacco. Overall, metal concentrations were only weakly intercorrelated (Table 2). Nickel and chromium concentrations were highly related (r = 0.60), but additional significant metal correlations were weak. When stratified by manufacturer, similar correlation trends were found, with nickel and chromium being significantly correlated for each Philip Morris, R.J. Reynolds and Other brands (r = 0.52, 0.546, and 0.565, respectively). Figure 1 presents box plots for metal contents by manufacturer, broken into 3 groups (Philip Morris USA (N = 112), RJ Reynolds (n = 112), Other Manufacturers (N = 87)). The Other Manufacturers group included Liggett Group (N = 16), Commonwealth Brands Inc. (N = 13), Lorillard (N = 11) and 20 other companies that each individually contributed less than 2% of the sample size. It can be seen that several samples fall outside 1.5 times the interquartile range. For example, sample 144 had extremely high levels of As, Cd, Cr, and Pb (but not Ni). Outliers can be identified in Table 3.

**Table 1:** Mean, Standard Deviation, and Range of Metal Concentration of Toxic Metals (micrograms per gram tobacco) (N = 311 except where noted).

Metals	Mean (μg·g^−1^)	Std. Deviation	Range
As	0.17	0.06	LOD–0.40
Cd	0.86	0.23	LOD–3.10
Cr (N = 310)	2.35	0.86	0.60–7.50
Ni	2.21	0.54	0.60–4.40
Pb (N = 310)	0.44	0.24	LOD–2.40

**Table 2:** Overall Metal Correlations and Metal Correlations by Manufacturer (micrograms per gram tobacco).

		Pb	Cd	As	Ni	Cr
Pb	Pearson Correlation	1	0.187 **	0.075	0.107	0.046
Cd	Pearson Correlation	0.187 **	1	0.088	0.136 *	0.081
As	Pearson Correlation	0.075	0.088	1	−0.114 *	0.021
Ni	Pearson Correlation	0.107	0.136 *	−0.114 *	1	0.597 **
Cr	Pearson Correlation	0.046	0.081	0.021	0.597 **	1
**Philip Morris**						
Pb	Pearson Correlation	1	0.108	0.225 *	0.130	0.095
Cd	Pearson Correlation	0.108	1	−0.045	0.135	−0.177
As	Pearson Correlation	0.225 *	−0.045	1	−0.095	−0.004
Ni	Pearson Correlation	0.130	0.135	−0.095	1	0.522 **
Cr	Pearson Correlation	0.095	−0.177	−0.004	0.522 **	1
**R.J. Reynolds**						
Pb	Pearson Correlation	1	−0.017	0.090	0.190 *	−0.060
Cd	Pearson Correlation	−0.017	1	−0.037	−0.217 *	−0.103
As	Pearson Correlation	0.090	−0.037	1	0.060	0.095
Ni	Pearson Correlation	0.190 *	−0.217 *	0.060	1	0.546 **
Cr	Pearson Correlation	−0.060	−0.103	0.095	0.546 **	1
**Other**						
Pb	Pearson Correlation	1	0.275 **	−0.043	0.115	0.112
Cd	Pearson Correlation	0.275 **	1	0.268 *	0.236 *	0.291 **
As	Pearson Correlation	−0.043	0.268 *	1	−0.172	0.080
Ni	Pearson Correlation	0.115	0.236 *	−0.172	1	0.565 **
Cr	Pearson Correlation	0.112	0.291 **	0.080	0.565 **	1

* Correlation is significant at the 0.05 level (2-tailed); ** Correlation is significant at the 0.01 level (2-tailed).

**Table 3:** Brand Outliers Identified in Boxplots of Metal Concentrations by Manufacturer (Figure 1).

Pack Number	Pack Name
12	Doral Ultra Light
27	Doral Menthol 100
42	Misty Menthol Green 120
54	Natural American Spirit Light
61	Basic Light
69	Marlboro Light
71	Marlboro Ultra Light 100
74	Benson and Hedges Menthol 100
81	Marlboro Light 100
82	Basic Light
85	Marlboro Light
100	Marlboro Light 100
106	Basic Light
112	Marlboro Ultra Light
115	Basic Ultra Light 100
118	Marlboro Light 100
129	Montclair Ultra Light
144	Poker Light 100
170	Virginia Slims Luxury Ultra Light 120
184	Main Street Menthol Light 100
214	Marlboro Ultra Light 100
216	Marlboro
222	Marlboro 100
246	Eve Ultra Light Slim 120
255	Ace Menthol Light 100
267	Merit Ultra Light
269	Marlboro King
273	disCOUNT Full Flavor 100
320	USA Gold FF 100

**Figure 2:**
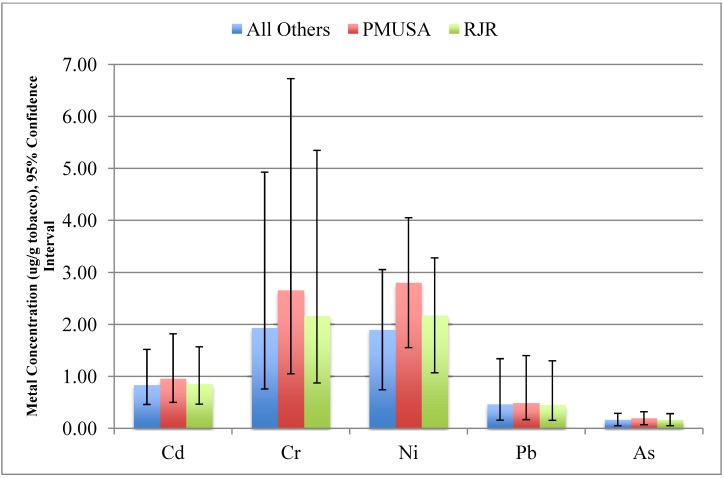
Adjusted mean metal concentration (μg·g^−1^ tobacco) by manufacturer.

Table 4 presents multivariate analyses controlling for product menthol status, Light/Mild labeling, per-cigarette tobacco weight, and moisture. Significant overall manufacturer differences were noted for Cd [Wald χ^2^ (2) =6.977, p = 0.031], Cr [Wald χ^2^ (2) = 37.849, p < 0.001], and Ni [Wald χ^2^ (2) = 59.388, p < 0.001]. Figure 2 illustrates the adjusted means by manufacturer. For Cd, post-hoc tests did not show pairwise differences between manufacturer groups (p’s >0.10). For Cr, RJR brands differed from OM brands (p = 0.02), and much more variety was seen in metal concentration for all brands. For Ni, we saw significant pairwise differences between PMUSA and RJR brands (p < 0.001), PMUSA and OM brands (p < 0.001), and RJR and OM brands (p = 0.006). Across manufacturers, product moisture was positively associated with Cr content (p = 0.004), and negatively associated with As content (p < 0.001). Light/Mild brands appeared to contain significantly less Ni than brands not so labeled (2.49 μg·g^−1^ *vs.* 2.09 μg·g^−1^, p = 0.02). 

**Table 4:** Regression (GEE) analysis of metals by product characteristics.

	As			Cd			Cr			Ni			Pb		
	B	SE	p	B	SE	p	B	SE	p	B	SE	p	B	SE	p
**(Intercept)**	0.347	0.0601	<0.001	0.243	0.216	0.261	−0.237	0.4311	0.583	1.92	0.5234	<0.001	−0.504	0.3838	0.189
**Other Manufacturer**	0.003	0.0097	0.791	−0.022	0.04	0.586	−**0.112**	0.0571	0.05	−**0.277**	0.0961	0.004	0.026	0.0736	0.720
**RJR**	0.028	0.0151	0.06	**0.111**	0.0461	0.017	**0.206**	0.0519	<0.001	**0.628**	0.1609	<0.001	0.075	0.102	0.463
**PMUSA**	REF			REF			REF			REF			REF		
**Menthol**	−0.005	0.01	0.612	0.008	0.0335	0.818	−0.051	0.0506	0.318	0.001	0.0991	0.99	0.197	0.1184	0.096
**Nonmenthol**	REF			REF			REF			REF			REF		
**Light/Mild descriptor**	0.006	0.0098	0.528	0.041	0.037	0.264	−0.039	0.0416	0.347	−**0.402**	0.1723	0.02	−0.003	0.073	0.966
**No descriptor**	REF			REF			REF			REF			REF		
**Per cigarette dry weight (g)**	0.014	0.0636	0.822	−0.2	0.2835	0.48	−0.249	0.2655	0.348	−0.638	0.4666	0.171	0.564	0.6339	0.374
**% moisture**	−**0.013**	0.0036	<0.001	−0.02	0.0189	0.29	**0.081**	0.0279	0.004	0.057	0.0339	0.093	−0.049	0.0336	0.142
**(Scale)**	0.005			0.056			0.662			0.271			0.059		

Finally, we estimated each individual’s potential exposure to each metal using the μg·g^−1^ concentration of each metal, the per-cigarette tobacco weight of their brand (mean = 0.62 g, SD = 0.08), their self-reported number of cigarettes smoked per day (mean = 19.6, SD = 8.0), and an estimate of transfer for each metal based on the midpoint of a range drawn from the literature [4]. Median values are presented in Table 5. In interpreting these data one must recognize that estimating daily exposure does not accurately translate into actual long term human exposure since most smokers smoke daily for decades. Even on an estimated daily exposure, however, we found that the median estimated availabilities of As, Cd, and Cr met or exceed the ‘no significant risk levels’ (NSRL) of those elements as defined by the State of California [38]. Indeed, all participants’ estimated exposures to Cd would exceed the NSRL, while 50% would exceed the NSRL for As. The estimated potential exposure to Pb is approximately 1/3 of the maximum daily dose (MADL), though 2.6% (N = 8) of cases would exceed this level. No comparable reference level exists for Ni. 

**Table 5:** Estimated median daily potential for exposure to metals based on self-reported cigarettes smoked per day, cigarette metal concentration (μg·g^−1^), and cigarette tobacco weight, as compared to California OEHHA safe harbor levels.

	% Transfer Range	Median Potential Daily Exposure (IQR)	Range	Cal OEHHA (μg/day, inhalation NSRL *^a^*)
**As**	0–7%	0.06 μg (0.05)	0–0.24	0.06 μg/day
**Cd**	7%–22%	1.36 μg (0.77)	0.46–6.48	0.05 μg/day
**Cr**	0.43%–1.74%	0.26 μg (0.18)	0.06–1.11	0.001 μg/day Cr(VI)
**Ni**	0.1%–2.4%	0.30 μg (0.15)	0.07–1.19	–
**Pb**	0.16%–6.3%	0.14 μg (0.10)	0–1.58	0.5 μg/day (MADL)

*^a^* [36]; IQR = interquartile range.

## 4. Conclusions

Cigarettes obtained from a sample of smokers in the United States were found to contain metals that could potentially be harmful to human health. The average metal concentrations observed per gram of unburned tobacco were comparable to amounts found in cigarettes on the US [8], Canadian [39], and Brazilian [21] markets, but less than those found in cigarettes purchased in China [22]. Recent data from the U.S. market [8] are particularly instructive; their data show a broadly similar pattern of findings.

In general, values in our study are slightly lower than those reported by Fresquez and colleagues; this could be due to the different time frames of collection (2009 *vs.* 2011), different mixes of products, and/or different analytical methods (XRF *vs.* ICP-MS). Analyses of reference tobaccos 2R4F and 3R4F analyzed by both laboratories are consistent, agreeing within their 2σ errors. The X-ray fluorescence method was preferred over more commonly applied methods such as ICP-MS or graphite furnace atomic absorption spectroscopy (GF-AA) in that no dissolution stage is required given that some metals are associated with highly refractory minerals, and a larger mass of sample can be analyzed to better represent the full blend of tobaccos in finished products. 

Cd in particular is regarded by the International Agency for Research on Cancer (IARC) as one of the “strong carcinogens” in tobacco smoke [40,41], with Cd, Ni, and As classified “carcinogenic to humans” (Class I). Metal levels associated with smoking are also associated with cancer incidence and mortality [42,43,44]. Studies show that metal concentrations are higher in pulmonary tissues of lung cancer cases than controls [45]; a similar pattern observed for head and neck cancers [46]. Among head and neck cancer cases, As and Cd levels in both tumor and healthy tissue samples were observed to be far higher in smokers than nonsmokers [46]. Furthermore, the ratio of rod to smoke concentrations of metals appears to increase when using the Canadian intense smoking protocol compared with the ISO protocol, suggesting greater transfer to smoke with increasing smoking intensity [39]. And while Jones *et al.* [47] report higher levels of Cd in blood of menthol smokers compared to nonmenthol smokers, we did not see a difference in terms of Cd content (p = 0.818) in our sample of brands, suggesting behavioral and/or absorption factors may better explain the difference in observed exposure. 

Other investigators have reported that counterfeit cigarettes have significantly higher levels of heavy metals than non-counterfeit cigarettes [23,24]. In the current study, cigarette samples were obtained directly from smokers, rather than through retail channels. The majority of participants reported that they purchased the pack sent from their usual outlet, the most common of which were convenience stores and tobacco discount outlets. We did note that nine subjects (2.5%) reported they had purchased the pack on the Internet or from another person, though we saw no conclusive evidence that any products were counterfeit. 

Our study is subject to a number of limitations. Packs were collected directly from consumers which has the advantage of reflecting the types of products that people use, although we do not have full knowledge of the chain-of-custody of the products sent to us for testing, so the results reported should not be treated as definitive for the brands tested. Still, our findings broadly mirror other published values. Second, while we estimated a potential smoke metal content and daily potential exposure from smoke from the data at hand, primarily to provide a sense of scale, we did not measure smoke concentrations directly. We also did not assess biomarkers of exposure to the toxicants (e.g., urinary metals), so we cannot make assessments of human body burden or health risk. 

Because of the variety of toxic heavy metals in cigarette tobacco, and the numerous side effects to the body that they cause, it is crucial that the FDA use its authority to encourage minimization of metal content in cigarette tobacco. Trace element patterns suggest various possible origins for the heavy metals including industrial pollution in growing regions and the misuse of fertilizers—regulation in these areas could decrease metal concentrations in legitimate cigarette products [23]. According to the World Health Organization Study Group on Tobacco Product Regulation (TobReg), further studies are required on the concentrations of metals in cigarette tobacco [48], as well as concentrations of metals in tobacco smoke obtained with the ISO Standard and Intense regimens in order to supplement current knowledge on the physical transport of metals into smoke. 

Regulatory recommendations by the TobReg include monitoring the tobacco blends in both combustible and noncombustible products offered for sale by requiring testing for levels of arsenic, cadmium, lead, and nickel by brand periodically and whenever the source of the tobacco shows substantial increases in the concentrations of any of the metals tested [48]. Although some have questioned whether monitoring of tobacco toxicants is potentially counterproductive for public health (e.g., [49,50]), limiting toxic constituents including metals to their lowest possible concentrations would constitute a reasonable interim regulatory measure to reduce the harms of tobacco given that FDA is prohibited from completing banning categories of tobacco products such as cigarettes. 
